# Changes in brainstem habituation during onabotulinumtoxinA treatment in chronic migraine: A prospective case–control study

**DOI:** 10.1111/head.15021

**Published:** 2025-07-15

**Authors:** Gerlinde Freimark, Sebastian Strauss, Lucas H. Overeem, Mira P. Fitzek, Kristin S. Lange, Carolin L. Höhne, Uwe Reuter, Robert Fleischmann, Bianca Raffaelli

**Affiliations:** ^1^ Department of Neurology Charité–Universitätsmedizin Berlin, corporate member of Freie Universität Berlin and Humboldt‐Universität zu Berlin Berlin Germany; ^2^ Department of Neurology University Medicine Greifswald Greifswald Germany; ^3^ Junior Clinician Scientist Program, Berlin Institute of Health at Charité Berlin Germany; ^4^ Clinician Scientist Program, Berlin Institute of Health at Charité Berlin Germany; ^5^ University Medicine Greifswald Greifswald Germany

**Keywords:** chronic migraine, disease modification, neurophysiology, onabotulinumtoxinA

## Abstract

**Objectives/Background:**

Chronic migraine is a debilitating neurological disorder characterized by central sensitization and impaired brainstem habituation. OnabotulinumtoxinA is an established prophylactic treatment for chronic migraine, yet its effects on central trigeminal sensory processing remain incompletely understood. The nociceptive blink reflex (nBR) is a well‐established neurophysiological tool for assessing brainstem excitability and central sensory processing within the trigeminal system. This prospective case–control study investigated longitudinal changes in brainstem neurophysiology following onabotulinumtoxinA treatment using the nBR.

**Methods:**

Between November 2022 and April 2024, we assessed nBR habituation in 27 patients with chronic migraine and compared them with 27 age‐ and sex‐matched healthy controls. Measurements were performed at peak efficacy (1 month postinjection, Month 1+) and prior to reinjection (3 months postinjection, Month 3+). Habituation of the polysynaptic R2 response was analyzed as the primary outcome.

**Results:**

At Month 1+, R2 nBR habituation in patients was similar to that observed in healthy controls (4‐s interstimulus interval, ipsilateral: *β* = 0.10, 95% confidence interval [CI] = −0.16 to 0.36, *p* = 0.457); however, by Month 3+, patients showed a significant impairment in R2 habituation compared to healthy controls (4‐s interstimulus interval, ipsilateral: *β* = −0.29, 95% CI = −0.55 to −0.03, *p* = 0.029). Correlation analyses revealed that reduced habituation was associated with increased monthly migraine days (4‐s interstimulus interval, contralateral: *r* = 0.41, 95% CI = 0.02 to 0.69, *p* = 0.039) and prolonged intervals since the last onabotulinumtoxinA treatment (4‐s interstimulus interval, Month 3+, ipsilateral: *r* = 0.33, 95% CI = 0.06 to 0.55, *p* = 0.018), which aligns with the clinical observation of wearing off. Supporting this notion, patients with more prior treatment cycles exhibited sustained improvement in habituation deficits (4‐s interstimulus interval, Month 3+, ipsilateral: *r* = −0.46, 95% CI = −0.71 to −0.09, *p* = 0.017). The monosynaptic R1 component remained unchanged between Month 1+ and 3+ (4‐s interstimulus interval, ipsilateral: *β* = −0.028, 95% CI = −0.067 to 0.011, *p* = 0.164), emphasizing a specific treatment effect of trigeminal system‐mediated pain processing at the brainstem level.

**Conclusion:**

These findings indicate that onabotulinumtoxinA exerts a neuromodulatory effect on brainstem neurophysiology, with R2 habituation improving during peak treatment efficacy and declining as the effect wears off. The results underscore the time‐dependent central effects of onabotulinumtoxinA on nociceptive processing within the trigeminal system. Future research should investigate the nBR as a potential biomarker for optimizing onabotulinumtoxinA treatment strategies in chronic migraine.

## Plain Language Summary

This study examines how onabotulinumtoxinA treatment affects brainstem function in people with chronic migraine. One month after treatment, patients’ brain responses to painful stimuli became more like those of healthy individuals, but this improvement faded by 3 months, consistent with the clinical wearing‐off effect. These findings suggest that onabotulinumtoxinA has an effect on brainstem pain processing, and further research could help optimize treatment timing for better migraine management.

**Abbreviations**
BoNT‐A: onabotulinumtoxinA
CGRP: calcitonin gene‐related peptide
CI: confidence interval
CM: chronic migraine
GLMM: generalized linear mixed model
IQR: interquartile range
mAb: monoclonal antibody
MHD: monthly headache days
MMD: monthly migraine days
nBR: nociceptive blink reflex
PREEMPT: Phase III Research Evaluating Migraine Prophylaxis Therapy
R1: R1 component of the nBR
R2: R2 component of the nBR

## INTRODUCTION

Migraine is a prevalent and disabling neurological disorder, affecting approximately 15% of the global population.^1^ Chronic migraine (CM) affects around 2% of the adult population^2^ and is defined by the occurrence of headaches on ≥15 days per month, with at least 8 days fulfilling criteria for migraine.^3^ CM significantly diminishes quality of life and imposes substantial societal and health care burdens, making effective treatment paramount.^4^, ^5^

One established treatment for the prevention of chronic migraine is onabotulinumtoxinA (BoNT‐A).^6^ Administered according to the Phase III Research Evaluating Migraine Prophylaxis Therapy (PREEMPT) protocol, this treatment involves 31 injections of 155 IU of BoNT‐A into specific head and neck muscles every 12 weeks, with up to 8 additional injections according to the follow‐the‐pain approach.^7^, ^8^, ^9^ Clinical trials and real‐world evidence have consistently demonstrated its efficacy in reducing headache frequency, severity, and associated disability.^7^, ^8^, ^9^, ^10^, ^11^, ^12^ BoNT‐A is generally well tolerated, with side effects such as neck pain and transient ptosis reported infrequently.^7^, ^8^, ^9^, ^13^ Maximum therapeutic efficacy within a single treatment cycle is typically observed 5–6 weeks postinjection.^14^ Long‐term use, often after the second or third cycle, results in sustained benefits for many patients.^13^ However, a significant proportion of patients experience a “wearing‐off” phenomenon, wherein the therapeutic effects diminish toward the end of the 12‐week treatment interval.^14^, ^15^

The mechanisms by which BoNT‐A mitigates migraine are multifaceted.^16^ At the peripheral level, BoNT‐A inhibits the release of pronociceptive neuropeptides such as calcitonin gene‐related peptide (CGRP) at trigeminal nerve endings.^17^ BoNT‐A may also exert effects on central trigeminovascular structures, potentially altering disease activity.^18^ A recent study suggests that BoNT‐A exerts a neuromodulatory effect by reducing input from trigeminovascular nociceptors and normalizing cortical activity, offering evidence of its impact on central pain processing mechanisms.^19^

Among the surrogate markers of central processing of trigeminal nociceptive input, the nociceptive blink reflex (nBR) is a well‐established electrophysiological tool.^20^ The nBR comprises an early, oligosynaptic ipsilateral R1‐component (R1) and two late, bilateral polysynaptic R2‐components (R2).^21^ The R2 response is primarily mediated by nociceptive afferents of the trigeminal nerve, which project through the tractus spinalis nervi trigemini and engage second‐order neurons within the brainstem.^22^, ^23^ This pathway is critical for nociceptive signal transmission and modulation at the brainstem level, providing insight into central sensitization mechanisms in chronic pain conditions and pain modulation.^24^ Patients with migraine frequently exhibit deficits in nBR habituation of the R2 response, reflecting impaired regulation of trigeminal nociceptive input and heightened brainstem excitability.^25^

This study aims to investigate the longitudinal effects of BoNT‐A on brainstem excitability in CM, as measured by the nBR. Specifically, we assess nBR habituation 1 and 3 months after BoNT‐A administration to determine whether the wearing‐off phenomenon correlates with changes in central trigeminal processing. Our primary hypothesis was that BoNT‐A treatment modulates brainstem excitability in CM, improving nBR R2 habituation at peak efficacy after 1 month, with a subsequent decline after 3 months, aligning with the expected wearing‐off effect. Additionally, we examine correlations between changes in nBR parameters and treatment efficacy. Understanding the temporal dynamics of brainstem excitability and its modulation by BoNT‐A could provide new insights into the central effects of this therapy and may help optimize treatment intervals on an individual basis.

## METHODS

### Study design and participants

The study was conducted between November 2022 and April 2024 at the Headache Center, Charité Universitätsmedizin Berlin, Berlin, Germany, as a prospective case–control study with longitudinal follow‐up, in collaboration with the University Medicine Greifswald, Germany. We included two groups of participants: (1) patients with CM who received BoNT‐A injections according to the PREEMPT protocol as part of their routine CM treatment and (2) a sex‐ and age‐matched healthy control group without migraine and without any BoNT‐A treatment. The patients with CM were recruited directly from the outpatient clinic of the Headache Center. The control participants were recruited via intranet announcements and direct contact. Direct contact was primarily initiated through private acquaintances of the study team. To minimize bias, we excluded individuals with a known professional or academic interest in headache or other neurological disorders.

This analysis is part of a larger prospective observational study investigating a range of biochemical and electrophysiological biomarkers during treatment with BoNT‐A in patients with CM. The present analysis of the nBR was defined a priori as primary neurophysiological endpoint of the study. No aspects of these data have been published previously.

### Inclusion and exclusion criteria

Participants in both groups were required to be between 18 and 65 years of age and to provide written informed consent prior to enrollment. They had to have no history of significant medical conditions that could interfere with the study outcomes, as judged by the investigators (e.g., severe psychiatric or neurological diseases). Pregnancy or breastfeeding also led to exclusion from the study.

For the migraine group, inclusion criteria required a diagnosis of CM classified according to the criteria of the International Classification of Headache Disorders, 3rd edition.^3^ Participants needed to have received BoNT‐A treatment approximately every 12 weeks with doses ranging from 155 to 195 IU, following the PREEMPT protocol. Although the PREEMPT protocol specifies exact 12‐week intervals, slightly extended intervals are standard in our clinical routine due to scheduling constraints or individual treatment decisions. Participants had to be undergoing BoNT‐A treatment for at least 3 months prior to enrollment, with no upper limit on treatment duration. Exclusion criteria for the migraine group included the use of concomitant prophylactic headache treatments beyond BoNT‐A or the presence of additional headache disorders. Concomitant medication for nonheadache indications was permitted, provided it remained stable for at least 3 months before inclusion and throughout the study duration.

For the healthy control group, participants were excluded if they had a history of chronic or recurrent headache disorders except for tension‐type headache occurring no more than 2 days per month.

### Study procedures

Suitable participants were screened and tested for eligibility. At screening, we collected demographic and anamnestic information, including age, sex, body mass index, comorbidities, and concomitant medication. In addition, all participants received a neurological and physical examination. Patients with migraine were asked to keep a standardized daily headache diary with information about headache characteristics, accompanying symptoms, and acute medication. A “headache day” was defined as any day with a reported headache, whereas a “migraine day” was classified as a day when the headache met the International Classification of Headache Disorders, 3rd edition criteria for probable migraine or triptan medication was used. We considered a month as the last 28 days preceding each study visit.

After the screening visit, participants attended two experimental study visits. For patients with migraine, one visit (Month 1+) was scheduled approximately 1 month after the most recent BoNT‐A injection, around the time of the expected maximum efficacy, whereas the second visit (Month 3+) occurred approximately 3 months postinjection, immediately before the next scheduled injection, when BoNT‐A efficacy was expected to be wearing off. The order of these visits was selected according to the patient's schedule.

For the healthy control group, the two visits were scheduled 1–2 months apart at random time points. This interval was selected to match the timeframe of the migraine group and ensure comparable temporal spacing between assessments.

Study visits for the migraine group were conducted exclusively during the interictal phase, defined as a migraine‐free period lasting at least 12 h before the visit. Participants were required to abstain from taking any pain medication within the 12 h preceding the visit. If a migraine attack occurred or if pain medication was taken within this time frame, the visit was canceled and rescheduled to ensure adherence to study criteria.

At every visit, after reviewing and assessing the participants’ headache diaries, we performed standardized measurements of the nBR, as described in detail below.

### Measurement of the nociceptive blink reflex

Stimulation and data acquisition were conducted using a commercial electrophysiology system (Keypoint, Natus Medical Incorporated, Middleton, WI, USA) with a bipolar montage of self‐adhesive surface electrodes. Electrical stimulation targeted the supraorbital branch of the trigeminal nerve on the affected side (matched in controls), and blink reflex responses were recorded bilaterally. In cases of bilateral or side‐shifting migraine attacks, we chose the right side by default. Pain threshold was determined using electrical stimuli applied at ≥30‐s intervals to prevent habituation. Subsequently, 60 stimuli (pulse width: 0.3 ms, intensity: 1.5 × pain threshold) were administered in six blocks of 10 stimuli. Given the influence of interstimulus interval on habituation, stimuli were delivered at three different interstimulus intervals (4 s, 9 s, 16 s) in a pseudorandomized order, with a ≥2‐min interblock interval to minimize habituation effects.^26^, ^27^, ^28^ Electromyographic traces were exported to MATLAB for preprocessing, following the algorithm by Thiele et al.^27^ The R1 component was identified within a 9–20‐ms poststimulus interval, whereas the R2 component was analyzed within a 30–80‐ms poststimulus window. The area of the R1 and R2 components was calculated using trapezoidal integration. Habituation was quantified as the beta coefficient (*β*_0_) derived from a linear regression model: *f*(R1*aᵢ*/R2*aᵢ*) *= β*_0_ *×* 2*aᵢ* + intercept (*i* = stimulus order). A negative slope (*β*_0_ < 0) indicated habituation, a positive slope (*β*_0_ > 0) reflected facilitation, and a slope of zero (*β*_0_ = 0) suggested no change in trigeminocervical complex responsiveness to repeated stimulation for R1 and R2, respectively.

### Endpoints

The primary endpoint of this study was the habituation of the nBR R2 following repeated stimulation at different time intervals between Month 1+ and Month 3+ in patients with migraine. Secondary endpoints included comparisons of the nBR R2 habituation between patients with migraine and healthy controls at different time points. Further exploratory analyses investigated correlations between nBR R2 habituation and several factors: changes in monthly headache days (MHD)/monthly migraine days (MMD) between Month 1+ and Month 3+, the number of BoNT‐A treatment cycles completed prior to study participation, and the number of days since the last BoNT‐A treatment. To assess potential influences of the local peripheral effects of BoNT‐A on muscle function and to specifically evaluate its impact on the oligosynaptic brainstem circuitry, as reflected by the R2 component of the nBR, habituation of the direct monosynaptic ipsilateral R1 response was also recorded.

### Sample size considerations and statistical analysis

The sample size calculation was performed using G*Power software, version 3.1.9.7.^29^ The original sample size calculation for the overall study was based on anticipated effect sizes for selected biochemical markers, which are not reported in this article. For the present analysis of the nBR, we evaluated whether the planned sample size of 27 patients would be sufficient to detect within‐subject changes in R2 habituation. Based on previous studies reporting changes in nBR habituation,^24^, ^30^, ^31^ we estimated that this sample size would provide >80% power to detect an at least comparable within‐subject effect (Cohen *dz* ≈ 0.7) using a paired‐sample design at a two‐tailed significance level of *α* = 0.05. Although the primary analysis in this study employed generalized linear mixed models (GLMMs), which more accurately model within‐subject variability and correlations, the simplified paired‐sample power analysis was used to demonstrate feasibility. Given the efficiency gains associated with GLMMs, the actual power is likely to be equal or higher than estimated with this conservative approach.

All statistical analyses were performed using R version 4.4.1 (R Foundation for Statistical Computing, Vienna, Austria) in the RStudio 2024.09.0 environment (Posit PBC, Boston, MA, USA). The packages Readxl (version 1.4.3), lme4 (version 1.1‐36), sjPlot (version 2.8.17), emmeans (version 1.10.7), dplyr (version 1.1.4), gt (version 0.11.1), mgcv (version 1.9‐1), performance (version 0.13.0), and lattice (version 0.22‐6) were used for model development and assessment. The distribution of continuous variables was visually assessed through inspection of histogram plots. Given their nonnormal distribution, continuous variables are reported as median (interquartile range [IQR]), and categorical variables are reported as count (percentage). Between‐group differences were assessed using the chi‐squared test or Mann–Whitney *U*‐test, as appropriate. To evaluate R2 habituation responses, we employed GLMMs fitted by maximum likelihood, which account for random effects and the correlation of repeated measures. Estimated marginal means and 95% confidence intervals (CI) were extracted for pairwise comparisons. Model assumptions for the GLMMs were evaluated by visual inspection of residual plots and quantile–quantile plots to assess normality, and by testing for homoscedasticity using a Levene‐type test on squared absolute residuals. Linearity was checked via residual versus outcome scatterplots and Pearson correlation. The random effects structure was assessed through model comparison using Akaike information criterion, and the final model was selected based on the lowest Akaike information criterion value. The negative values were adjusted with a standard value to enable model fitting and subtracted afterward for interpretability.

Pearson correlation coefficients and their corresponding 95% CIs were calculated using Fisher z‐transformation, as implemented in the cor.test() function in R. Pearson correlation analyses were conducted to assess whether changes in R2 nBR habituation responses were associated with changes in MHD and MMD. Additionally, we examined correlations between the number of prior BoNT‐A treatment cycles and R2 nBR habituation responses at each time point, as well as the time in days since the last treatment and R2 nBR habituation responses. Generalized additive models were used as a sensitivity analysis for the Pearson correlation.

All statistical tests were conducted using a two‐tailed significance level of *α* = 0.05.

### Standard protocol approvals, registrations, and patient consents

The Charité Ethical Committee approved the study protocol (EA2/196/23). All participants received detailed verbal and written information about the study procedures, potential risks, and data use, and subsequently provided written informed consent prior to participation.

## RESULTS

### Demographics and clinical response

This study included a total of 54 participants. Of 32 initially screened patients with CM, 27 were included in this analysis. One patient discontinued BoNT‐A treatment before the second experimental visit, and four were excluded due to missing nBR data. The control group consisted of 27 age‐ and sex‐matched healthy participants. There were no missing or excluded data in the final dataset. An overview of the demographic characteristics of all participants is provided in Table 1.

**TABLE 1:** Demographic characteristics of study population.

Demographic	Healthy controls, *n* = 27	Migraine patients, *n* = 27	*p*
Age, years	46.0 (20.0)	45.0 (16.5)	0.966
Female	20 (74)	20 (74)	>0.999
Body mass index^a^	24.6 (3.5)	27.4 (8.2)	0.261
Aura	–	8 (29)	n.a.
Disease duration, years	–	26.0 (20.5)	n.a.
Number of prior BoNT‐A treatment cycles	–	6.0 (11.5)	n.a.

*Note*: Values are given in median (interquartile range) or *n* (%).

Abbreviations: BoNT‐A, onabotulinumtoxinA; n.a., not applicable.

^a^ Calculated as kg/m^2^.

The first measurement (Month 1+) was conducted 28 (IQR = 3) days following BoNT‐A treatment; the second measurement (Month 3+) occurred 96 (IQR = 6) days after treatment. During Month 1+, patients reported a median of 7.5 (IQR = 7.5) MHD, of which 7.0 (IQR = 7.8) were MMD. By Month 3+, these increased to 9.0 (IQR = 8.5) MHD and 7.5 (IQR = 9.0) MMD, respectively, although the differences did not reach statistical significance (Table 2).

**TABLE 2:** Headache characteristics and pain thresholds of study population.

	Month 1+	Month 3+	*p*
Headache characteristics [patients]			
Last BoNT‐A treatment cycle, days	28 (3)	96 (6)	n.a.
Monthly headache days prior to visit	7.5 (7.5)	9.0 (8.5)	0.148
Monthly migraine days prior to visits	7.0 (7.8)	7.5 (9.0)	0.228
Acute medication days	4.0 (5.0)	5.0 (6.5)	0.069
Pain threshold, mA			
Controls	11.2 (5.5)	13.0 (5.0)	0.313
Migraine patients	11.0 (4.6)	12.0 (5.3)	0.819

*Note*: Values are given in median (interquartile range).

Abbreviations: BoNT‐A, onabotulinumtoxinA; n.a., not applicable.

There was no significant difference in the mean pain threshold after supraorbital stimulation between patients with migraine and controls at Month 1+ (*p* = 0.828) and Month 3+ (*p* = 0.338). Additionally, the pain threshold did not change between Month 1+ and Month 3+ for either group (controls, *p* = 0.313; patients with migraine, *p* = 0.819; Table 2).

### Comparison of R2 nBR habituation in patients with migraine and controls

In the GLMM analysis, a significant interaction effect between group and time was observed for R2 nBR habituation on the ipsilateral side with a 4‐s interstimulus interval (*β* = 0.39, 95% CI = 0.21 to 0.57, *p* < 0.001; Table 3). Within‐group analyses showed that in patients with migraine, nBR habituation significantly decreased from Month 1+ to Month 3+ (*β* = −0.28, 95% CI = −0.41 to −0.15, *p* < 0.001), whereas no significant change was observed in controls (*β* = 0.11, 95% CI = −0.01 to 0.24, *p* = 0.079; Table 3). At Month 3+, patients with migraine demonstrated significantly lower habituation than controls (*β* = −0.29, 95% CI = −0.55 to −0.03, *p* = 0.029), whereas at Month 1+, no significant difference between groups was found (*β* = 0.10, 95% CI = −0.16 to 0.36, *p* = 0.457).

**TABLE 3:** Estimated effects of group, time, and group × time interaction on R2 habituation of the nBR at a 4‐s interstimulus interval.

R2 nBR habituation	Interstimulus interval 4 s			
	Ipsilateral		Contralateral	
Predictors	Estimate (95% CI)	*p*	Estimate (95% CI)	*p*
Intercept	−0.74 (−0.93 to −0.55)	<0.001	−0.47 (−0.73 to −0.21)	<0.001
Group	−0.10 (−0.36 to 0.16)	0.459	−0.23 (−0.59 to 0.14)	0.215
Time	−0.11 (−0.24 to 0.01)	0.082	−0.08 (−0.27 to 0.10)	0.380
Group × time	0.39 (0.21 to 0.57)	**<0.001**	0.26 (−0.01 to 0.52)	0.056
Random effects				
*σ* ^2^ [residual variance]	<0.01		0.01	
*τ*_00_ [ID variance]	0.07 ID		0.13 ID	
ICC [intraclass correlation]	0.94		0.94	
*N* [participants]	54 ID		54 ID	
Marginal R2/conditional R2	0.154/0.949		0.048/0.943	
Within‐group differences				
Group	Estimate (95% CI)	*p*	Estimate (95% CI)	*p*
Control	0.11 (−0.01 to 0.24)	0.079	0.08 (−0.1 to 0.27)	0.378
Migraine	−0.28 (−0.41 to −0.15)	**<0.001**	−0.17 (−0.35 to 0.01)	0.062
Between‐group differences				
Time	Estimate (95% CI)	*p*	Estimate (95% CI)	*p*
Month 1+	0.10 (−0.16 to 0.36)	0.457	0.23 (−0.13 to 0.59)	0.212
Month 3+	−0.29 (−0.55 to −0.03)	**0.029**	−0.03 (−0.39 to 0.34)	0.889

*Note*: Generalized Linear Mixed Models fitted by maximum likelihood. Bold values indicate statistical significance.

Abbreviations: CI, confidence interval; ID, subject identifier; nBR, nociceptive blink reflex.

The GLMM of R2 nBR habituation at the contralateral side with a 4‐s interstimulus interval revealed a marginally significant interaction between group and time (*β* = 0.26, 95% CI = −0.01 to 0.52, *p* = 0.056). Post hoc within‐group analyses showed no significant changes in R2 nBR habituation from Month 1+ to Month 3+ in either patients with migraine or controls (Table 3). Similarly, post hoc pairwise comparisons indicated no significant differences between groups at either time point.

**FIGURE 1:**
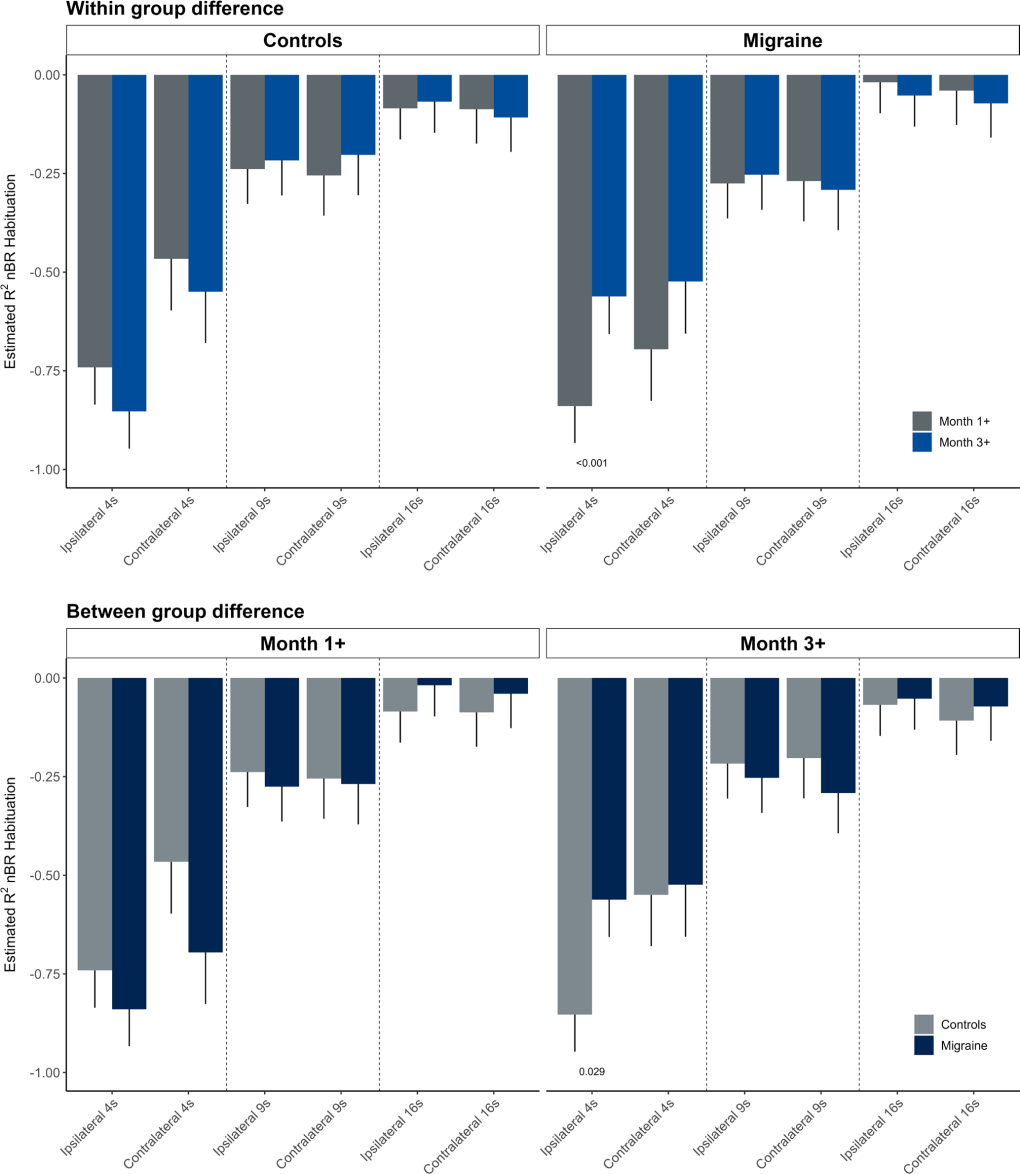
Estimated within‐ and between‐group differences in R2 habituation of the nociceptive blink reflex (nBR). Top panel: Estimated within‐group changes in R2 habituation at a 4‐s interstimulus interval are shown for healthy controls and patients with chronic migraine receiving onabotulinumtoxinA treatment. Measurements are shown at Month 1+ (gray) and Month 3+ (blue), representing peak and waning treatment phases, respectively. Bottom panel: Estimated between‐group comparisons in *R* ^2^ habituation at Month 1+ and Month 3+ between healthy controls (gray) and patients with migraine (blue). [Color figure can be viewed at wileyonlinelibrary.com]

The GLMM of R2 nBR habituation at the ipsilateral and contralateral sides with an interstimulus intervals of 9 s and 16 s revealed no significant effects, with stable outcomes across groups and time (Tables S1 and S2). The adjustment for the pain threshold did not significantly change the effect of any of our models (*p* > 0.05; data not shown). All estimates for R2 nBR habituation are presented in Figure 1.

Post hoc exploratory analyses comparing male and female participants indicated that the observed impairment in R2 habituation over time was primarily driven by the female subgroup. In contrast, male participants exhibited relatively stable or even improved habituation between time points (Tables S3–S5). However, these findings should be interpreted with caution given the limited number of male participants (*n* = 7). Further post hoc exploratory analyses revealed no significant differences between patients with (*n* = 7) and without (*n* = 20) aura (Tables S6–S8).

In contrast to the observed changes in R2 nBR habituation, the ipsilateral R1 nBR habituation remained stable over time in patients with migraine (*β* = −0.028, 95% CI = −0.067 to 0.011, *p* = 0.164 at a 4‐s interstimulus interval; *β* = 0.009, 95% CI = −0.030 to 0.048, *p* = 0.641 at 9 s; *β* = −0.003, 95% CI = −0.038 to 0.032, *p* = 0.876 at 16 s). Moreover, no significant differences in R1 nBR habituation were found between patients with migraine and controls at any time point or interstimulus interval (*p* > 0.1 for all comparisons).

### Correlations between R2 nBR habituation and headache parameters

**FIGURE 2:**
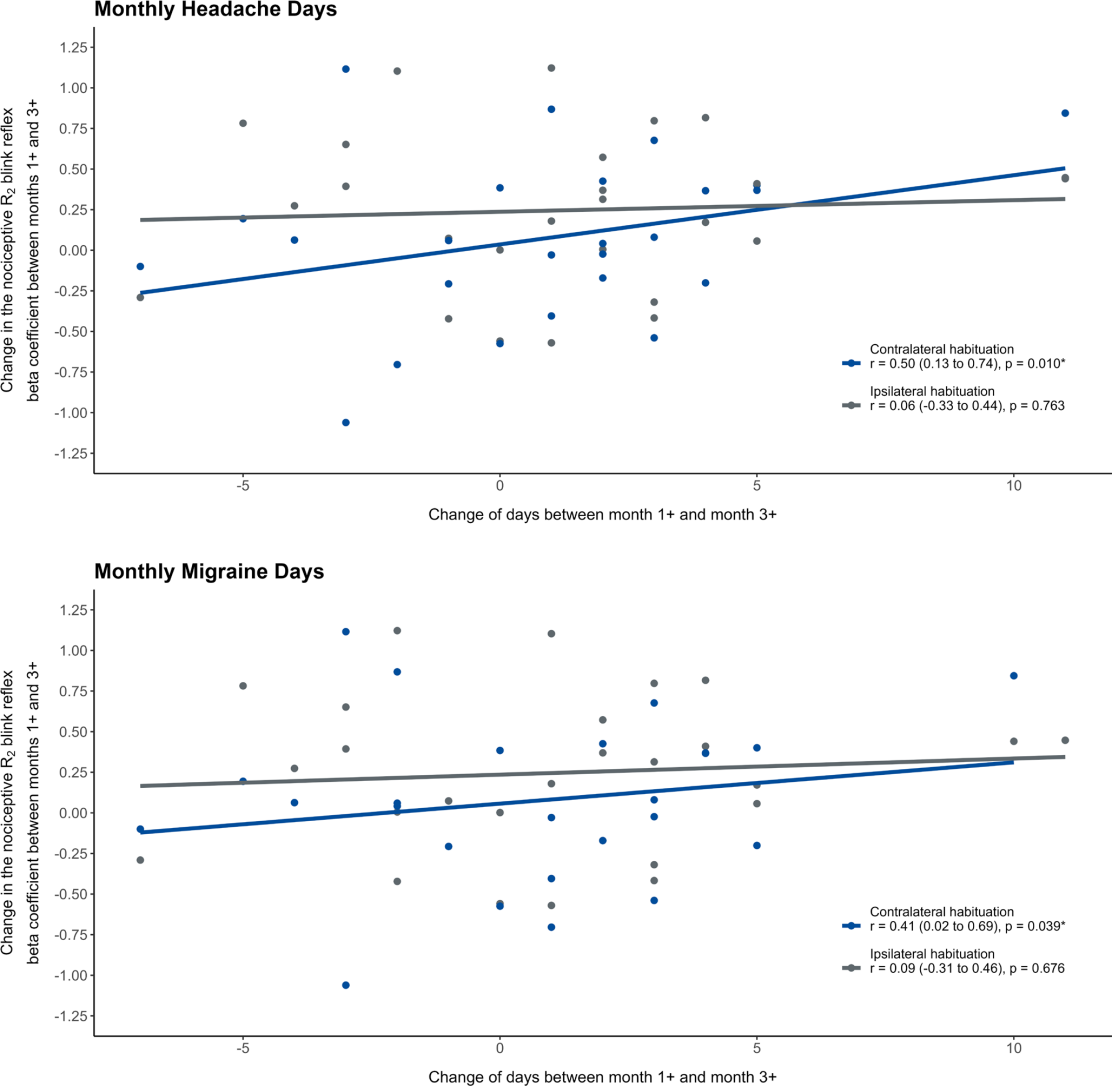
Correlation between changes in R2 habituation of the nociceptive blink reflex (nBR) and changes in headache/migraine days. The figure illustrates correlations between the change in R2 habituation (beta coefficient) of the nBR at a 4‐s interstimulus interval and the change in monthly headache days (top panel) and monthly migraine days (bottom panel) from Month 1+ (peak treatment effect) to Month 3+ (treatment waning). A significant negative correlation was observed on the contralateral side (blue) for both headache days (*r* = −0.56, *p* = 0.010) and migraine days (*r* = −0.45, *p* = 0.039), indicating that greater improvement in habituation was associated with stronger clinical response. No significant correlation was found on the ipsilateral side (gray). Statistically significant differences are marked with an asterisk. [Color figure can be viewed at wileyonlinelibrary.com]

Changes in R2 nBR habituation at a 4‐s interstimulus interval between Month 1+ and Month 3+ were significantly correlated with changes in MHD (*r* = 0.50, 95% CI = 0.13 to 0.74, *p* = 0.010) and MMD (*r* = 0.41, 95% CI = 0.02 to 0.69, *p* = 0.039) at the contralateral side, but not at the ipsilateral side (Figure 2). This indicates that a reduction in MHD or MMD is associated with an improvement in R2 nBR habituation (i.e., lower habituation values), whereas an increase in MHD or MMD corresponds to an impaired habituation. No significant associations were observed at other interstimulus intervals.

**FIGURE 3:**
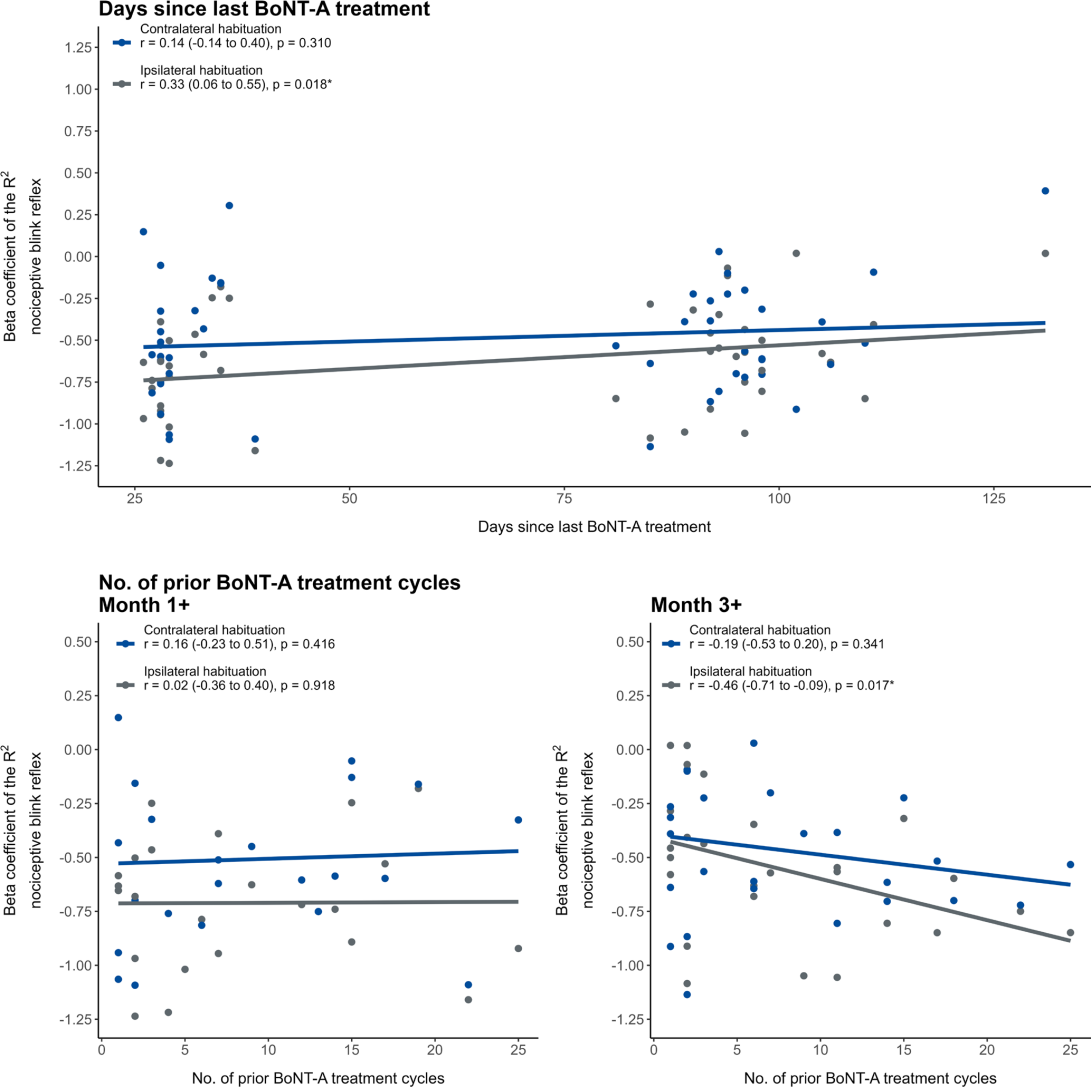
Association between onabotulinumtoxinA (BoNT‐A) treatment history and R2 habituation of the nociceptive blink reflex (nBR). Top panel: Scatterplot showing the relationship between the number of days since the most recent BoNT‐A injection and the R2 habituation (beta coefficient) of the nBR, indicating how the neurophysiological response changes as the treatment effect wears off. Bottom panels: Correlation between the number of prior BoNT‐A treatment cycles and the R2 habituation at Month 1+ (left) and Month 3+ (right). Statistically significant differences are marked with an asterisk. [Color figure can be viewed at wileyonlinelibrary.com]

Additionally, a significant positive correlation was observed between the number of days since the last BoNT‐A treatment and ipsilateral R2 nBR habituation at a 4‐s interstimulus interval (*r* = 0.33, 95% CI = 0.06 to 0.55, *p* = 0.018; Figure 3). This indicates that the impairment of R2 nBR habituation becomes more pronounced as the time since the last BoNT‐A treatment increases.

Regarding the number of prior BoNT‐A treatment cycles, a significant negative correlation was found with ipsilateral R2 nBR habituation at a 4‐s interstimulus interval at Month 3+ (*r* = −0.46, 95% CI = −0.71 to −0.09, *p* = 0.017; Figure 3). In contrast, no significant correlation was observed at Month 1+ (*r* = 0.02, 95% CI = −0.36 to 0.40, *p* = 0.918) or with other interstimulus intervals. This suggests that the habituation deficit at Month 3+ is less pronounced with increasing number of treatment cycles, that is, longer treatment duration.

The results from the generalized additive model largely confirmed the findings from the primary analysis, with significant effects observed in the same conditions (e.g., 4‐s interstimulus interval at the contralateral side for MHD/MMD and ipsilateral side for BoNT‐A treatment cycles and days since last treatment). However, the explained variance in this model remained relatively low (20%), indicating that additional factors may contribute to changes in R2 nBR habituation.

## DISCUSSION

This study investigated the effects of BoNT‐A treatment on nBR habituation in patients with CM over time. Significant alterations of the R2 habituation were observed 3 months post‐BoNT‐A injection compared to the 1‐month follow‐up, whereas the monosynaptic R1 component remained unchanged. This finding suggests a time‐dependent modulation of brainstem excitability and nociceptive processing under BoNT‐A treatment. After 1 month, during the expected peak efficacy of BoNT‐A, R2 nBR habituation was comparable to that of healthy control participants. In contrast, after 3 months, R2 nBR habituation was significantly impaired, coinciding with a slight decline in therapeutic efficacy. This pattern suggests that the neuromodulatory impact of BoNT‐A diminishes over time, consistent with the clinical observation of the “wearing‐off” phenomenon commonly reported by patients.^14^

The neuromodulatory effects of BoNT‐A in CM have been previously explored in the study by Sebastianelli et al., which demonstrated increased nBR R2 habituation on the contralateral side after a single session of BoNT‐A injections compared to baseline prior to treatment initiation.^19^ Our findings build upon these results by examining nBR habituation under sustained BoNT‐A treatment in a larger cohort with matched healthy controls. Consistent with these previous observations, the nBR R2 habituation of patients with CM 1 month after BoNT‐A treatment—corresponding to the expected peak of treatment efficacy—was similar to that observed in healthy controls, whereas an altered habituation pattern was observed at Month 3+. Given the absence of pretreatment baseline measurements in our cohort, it is not possible to definitely conclude that BoNT‐A normalized habituation. Instead, our findings suggest a transient modulation of brainstem excitability during the effective treatment window.

In our study, significant changes between Month 1+ and Month 3+ were observed ipsilaterally and only at the 4‐s interval, whereas no significant effects were detected at longer intervals of 9 and 16 s. This disparity may reflect the differential sensitivity of shorter interstimulus intervals in capturing changes in brainstem excitability.^26^, ^32^ Shorter intervals challenge the nociceptive system's capacity for sustained processing and habituation, making them a more sensitive marker of subtle neuromodulatory effects. Conversely, longer intervals may allow for partial recovery of baseline excitability,^33^ potentially masking the dynamic changes induced by BoNT‐A. The impaired habituation observed after >12 weeks posttreatment underscores the time‐dependent nature of BoNT‐A's effects. This finding is consistent with a wearing‐off phenomenon, in which both clinical and neurophysiological benefits gradually diminish toward the end of the treatment cycle. In line with this, we observed a significant correlation between impaired habituation and the time elapsed since the last BoNT‐A injection.

An important question arising from these findings is whether BoNT‐A exerts a disease‐modifying effect or whether the observed changes are primarily a consequence of the reduction in migraine days. Although we found a modest correlation between changes in migraine frequency and nBR habituation, the explained variance was small, suggesting that BoNT‐A may directly modify central pain processing mechanisms, extending beyond its symptomatic relief.^34^, ^35^ Similar findings have been reported in a study investigating CGRP monoclonal antibodies (mAbs), which demonstrated that treatment restored impaired brainstem habituation in patients with episodic migraine.^27^ Significant improvements in nBR R2 parameters were observed after 3 months of treatment with CGRP antibodies, with normalization of habituation to levels comparable to healthy controls.^27^ These observations parallel our findings, as both therapies appear to affect central nociceptive processing, suggesting that their effects are not limited to peripheral mechanisms but include secondary central adaptations.

The mechanisms underlying BoNT‐A's therapeutic effects in migraine are complex and multifaceted, extending well beyond its primary action of inhibiting acetylcholine release at neuromuscular junctions.^36^ Although this effect contributes to muscle relaxation, its efficacy in migraine prevention stems from additional mechanisms.^37^, ^38^ The stability of the monosynaptic R1 also argues against a pure peripheral neuromuscular effect, indicating that BoNT‐A does not affect the early, direct blink reflex pathway but rather modulates brainstem circuits involved in R2 processing. BoNT‐A inhibits the release of proinflammatory neurotransmitters^39^ and neuropeptides such as CGRP,^40^, ^41^, ^42^ which plays a pivotal role in migraine pathophysiology by promoting vasodilation, neurogenic inflammation, and nociceptive transmission.^43^ It also prevents the insertion of pain‐related ion channels and receptors, including TRPV1 and TRPA1, into neuronal membranes, which are critical mediators of peripheral and central sensitization.^44^, ^45^ Furthermore, BoNT‐A may modulate inflammatory gene expression, reducing neurogenic inflammation and altering immune cell activity.^46^ In our study, the pattern of nBR habituation observed during the peak efficacy period of BoNT‐A treatment may reflect a transient reduction in trigeminovascular sensitization, potentially leading to enhanced antinociceptive activity within the brainstem and improved central pain modulation.

In addition to these mechanisms, sex hormones may also play a modulatory role in brainstem excitability. Estrogens and progesterone are capable of crossing the blood–brain barrier and have been shown to influence central pain processing.^47^ Interestingly, in our exploratory analyses, the impairment of nBR habituation over time was observed only in female participants. Although this finding should be interpreted with caution due to the small number of male participants and limited statistical power, it highlights the need for future research to systematically investigate the influence of sex hormones and hormonal fluctuations on brainstem neurophysiology in migraine. Moreover, the potential impact of aura status on nBR habituation should also be evaluated in adequately powered studies.

The observed wearing‐off phenomenon, evidenced by a reduction in nBR habituation at Month 3+, suggests that the neuromodulatory effects of BoNT‐A diminish as peripheral sensitization recurs. These findings underscore the importance of adhering to 12‐week intervals for BoNT‐A injections to sustain its therapeutic benefits and minimize the recurrence of central sensitization.^48^, ^49^ Notably, we observed a negative correlation between the number of prior BoNT‐A treatment sessions and impaired habituation, suggesting that prolonged use of BoNT‐A may lead to cumulative and sustained neuromodulatory effects. This aligns with long‐term clinical studies demonstrating continuous improvement in migraine outcomes with BoNT‐A over time.^50^ Similarly, studies on CGRP mAbs have shown progressively improving efficacy over 3 years,^51^, ^52^ supporting the potential for sustained neuromodulatory benefits with prolonged treatment.

Finally, the potential of nBR as a biomarker for treatment optimization deserves further exploration. As previously suggested for CGRP mAbs,^27^ nBR assessments could help tailor treatment intervals and guide decisions about treatment continuation or cessation. Similarly, changes in R2 nBR habituation could serve as a tool to identify patients who might benefit from extended BoNT‐A intervals or additional injections, enabling personalized migraine management strategies.

A key strength of this study is the inclusion of a well‐defined cohort of patients with CM and age‐ and sex‐matched healthy controls. Additionally, the longitudinal design enabled the assessment of temporal dynamics in brainstem excitability across predefined time points. The main limitation of the study is the absence of baseline measurements prior to the initiation of BoNT‐A treatment, which prevents direct evaluation of changes relative to the untreated state. As a result, it is not possible to determine whether the neurophysiological patterns observed at Month 1+ represent a true normalization of nBR habituation or simply a transient modulation during peak treatment efficacy. Another limitation is the potential influence of confounders such as comorbidities and concomitant medications, which might have contributed to the observed findings. The recruitment of health care professionals and private acquaintances as healthy controls may have introduced a selection bias, despite efforts to exclude individuals with a specific interest in headache or other neurological disorders. The generalizability of our findings is limited by the specific study population, which included only patients with CM undergoing stable treatment with BoNT‐A, recruited from a single tertiary care center. Our strict inclusion and exclusion criteria may further limit external applicability. In addition, the study lacked a CM control group not receiving BoNT‐A, which limits our ability to attribute the observed neurophysiological changes specifically to the treatment rather than to other time‐related or disease‐inherent factors. Moreover, although our findings suggest a modulation of central neurophysiological mechanisms, the study design does not allow us to definitively distinguish whether these effects reflect direct central action of BoNT‐A or secondary adaptations following symptomatic relief. Furthermore, as our study focuses on interictal measurements, the lack of ictal assessments means that changes in habituation during acute migraine attacks remain unexplored. Additionally, the investigator who measured the nBR (G.F.) was not blinded to study group or sequence, which may introduce bias; however, the neurophysiology traces were analyzed by another investigator (S.S.) according to a predefined script to mitigate this limitation. Finally, exploring nBR parameters in individuals undergoing BoNT‐A treatment for nonmigraine indications, such as aesthetic purposes, could help differentiate the specific neuromodulatory effects of BoNT‐A in migraine versus its broader physiological impacts.

In conclusion, this study provides novel insights into the temporal dynamics of BoNT‐A's effects on brainstem excitability in patients with CM. Our findings highlight a transient modulation of nBR habituation during the peak efficacy period of BoNT‐A, suggesting a central neuromodulatory effect that appears to diminish as treatment effects wear off. These results support the potential for long‐term neuromodulatory benefits and raise questions about BoNT‐A's disease‐modifying capacity. Future research should focus on investigating the utility of nBR as a biomarker for optimizing personalized treatment strategies, ultimately aiming to improve outcomes for patients with CM.

## AUTHOR CONTRIBUTIONS

**Gerlinde Freimark:** Data curation; investigation; writing – original draft. **Sebastian Strauss:** Data curation; formal analysis; investigation; writing – original draft. **Lucas H. Overeem:** Data curation; formal analysis; supervision; visualization; writing – original draft. **Mira P. Fitzek:** Investigation; writing – review and editing. **Kristin S. Lange:** Investigation; writing – review and editing. **Carolin L. Höhne:** Investigation; writing – review and editing. **Uwe Reuter:** Supervision; writing – review and editing. **Robert Fleischmann:** Conceptualization; methodology; supervision; writing – review and editing. **Bianca Raffaelli:** Conceptualization; data curation; methodology; project administration; supervision; writing – original draft.

## CONFLICT OF INTEREST STATEMENT

**Gerlinde Freimark** declares no conflicts of interest. **Sebastian Strauss** reports personal fees from Lundbeck, Teva, and Novartis. **Lucas H. Overeem** declares no conflicts of interest. **Mira P. Fitzek** reports personal fees from Teva and Novartis. **Kristin S. Lange** reports a research grant from the International Headache Society and personal fees from Teva and Organon. **Carolin L. Höhne** declares no conflicts of interest. **Uwe Reuter** reports institutional fees from Allergan, AbbVie, Eli Lilly, Lundbeck, Novartis, Pfizer, Medscape, StreaMedUp, Springer, and Teva and research funding from Novartis. **Robert Fleischmann** reports personal fees from AbbVie and Novartis. **Bianca Raffaelli** reports research grants from Lundbeck, Novartis, the German Research Foundation, and Else Kröner‐Fresenius‐Stiftung and personal fees from AbbVie/Allergan, Eli Lilly, Lundbeck, Novartis, Organon, Perfood, and Teva.

## Supporting information


**Data S1.** Supporting information.

## Data Availability

Data not included in the article due to space limitations will be provided anonymized upon reasonable request to the corresponding author.
