# Supplementary material for: Changes in brainstem habituation during onabotulinumtoxinA treatment in chronic migraine: A prospective case–control study
Source: Headache. 2025 Jul 15;66(4):950–62. doi: 10.1111/head.15021 (PMC13044560; doi:10.1111/head.15021)
Supplement: Supplementary file 1 — Data S1. Supporting information. [file HEAD-66-950-s001.docx]

**Supplemental Table 1: Comparisons of R2 nBR habituation change over time and between migraine patients and controls with an interstimulus interval of 9s**

| **R² nBR Habituation** | **Interstimulus Interval 9s** | | | |
| --- | --- | --- | --- | --- |
|  | **Ipsilateral** | | **Contralateral** | |
| **Predictors** | **Estimate (95% CI)** | **p** | **Estimate (95% CI)** | **p** |
| (Intercept) | -0.24 (-0.41 to -0.06) | <0.001 | -0.26 (-0.46 to -0.05) | <0.001 |
| Group | -0.04 (-0.28 to 0.21) | 0.769 | -0.01 (-0.30 to 0.27) | 0.923 |
| Time | 0.02 (-0.13 to 0.17) | 0.774 | 0.05 (-0.13 to 0.23) | 0.564 |
| Group × Time | 0.00 (-0.21 to 0.21) | 0.996 | -0.07 (-0.33 to 0.18) | 0.557 |
|  | | | | |
| **Random Effects**: | | | | |
| σ² (Residual Variance) | <0.01 | | 0.01 | |
| τ₀₀ (ID Variance) | 0.06 ID | | 0.07 ID | |
| ICC (Intraclass Correlation) | 0.93 | | 0.92 | |
| N (Participants) | 54 ID | | 54 ID | |
| Marginal R² / Conditional R² | 0.008 / 0.929 | | 0.014 / 0.923 | |
|  | | | | |
| **Between Group Differences**: | | | | |
| **Time** | **Estimate (95% CI)** | **p** | **Estimate (95% CI)** | **p** |
| Month 1+ | 0.04 (-0.21 to 0.28) | 0.768 | 0.01 (-0.27 to 0.30) | 0.923 |
| Month 3+ | 0.04 (-0.21 to 0.28) | 0.772 | 0.09 (-0.19 to 0.37) | 0.538 |
|  | | | | |
| **Within Group Differences**: | | | | |
| **Group** | **Estimate (95% CI)** | **p** | **Estimate (95% CI)** | **p** |
| Control | -0.02 (-0.17 to 0.13) | 0.773 | -0.05 (-0.23 to 0.12) | 0.563 |
| Migraine | -0.02 (-0.17 to 0.13) | 0.767 | 0.02 (-0.15 to 0.20) | 0.800 |
| **Note:** Generalized Linear Mixed Models (GLMMs) fitted by maximum likelihood. | | | | |

**Supplemental Table 2: Comparisons of R2 nBR habituation change over time and between migraine patients and controls with an interstimulus interval of 16s**

| **R² nBR Habituation** | **Interstimulus Interval 16s** | | | |
| --- | --- | --- | --- | --- |
|  | **Ipsilateral** | | **Contralateral** | |
| **Predictors** | **Estimate (95% CI)** | **p** | **Estimate (95% CI)** | **p** |
| (Intercept) | -0.08 (-0.24 to 0.07) | <0.001 | -0.09 (-0.26 to 0.09) | <0.001 |
| Group | 0.07 (-0.15 to 0.29) | 0.553 | 0.05 (-0.20 to 0.29) | 0.701 |
| Time | 0.02 (-0.09 to 0.13) | 0.764 | -0.02 (-0.17 to 0.12) | 0.775 |
| Group × Time | -0.05 (-0.21 to 0.11) | 0.527 | -0.01 (-0.22 to 0.19) | 0.913 |
|  | | | | |
| **Random Effects**: | | | | |
| σ² (Residual Variance) | <0.01 | | <0.01 | |
| τ₀₀ (ID Variance) | 0.05 ID | | 0.06 ID | |
| ICC (Intraclass Correlation) | 0.95 | | 0.93 | |
| N (Participants) | 54 ID | | 54 ID | |
| Marginal R² / Conditional R² | 0.012 / 0.952 | | 0.010 / 0.934 | |
|  | | | | |
| **Between Group Differences**: | | | | |
| **Time** | **Estimate (95% CI)** | **p** | **Estimate (95% CI)** | **p** |
| Month 1+ | -0.07 (-0.28 to 0.15) | 0.551 | -0.05 (-0.29 to 0.19) | 0.701 |
| Month 3+ | -0.02 (-0.23 to 0.20) | 0.889 | -0.04 (-0.28 to 0.21) | 0.770 |
|  | | | | |
| **Within Group Differences**: | | | | |
| **Group** | **Estimate (95% CI)** | **p** | **Estimate (95% CI)** | **p** |
| Control | -0.02 (-0.13 to 0.09) | 0.764 | 0.02 (-0.12 to 0.16) | 0.775 |
| Migraine | 0.03 (-0.08 to 0.15) | 0.551 | 0.03 (-0.11 to 0.18) | 0.661 |
| **Note:** Generalized Linear Mixed Models (GLMMs) fitted by maximum likelihood. | | | | |

**Supplemental Table 3: Comparisons of R2 nBR habituation change over time and between male and female patients with migraine with an interstimulus interval of 4s**

| **R² nBR Habituation** | **Interstimulus Interval 4s** | | | |
| --- | --- | --- | --- | --- |
|  | **Ipsilateral** | | **Contralateral** | |
| **Predictors** | **Estimate (95% CI)** | **p** | **Estimate (95% CI)** | **p** |
| (Intercept) | -0.64 (-1.01 to -0.27) | <0.001 | -0.41 (-0.41 to -0.4) | <0.001 |
| Sex | -0.28 (-0.7 to 0.15) | 0.201 | -0.38 (-0.38 to 0.37) | <0.001 |
| Time | -0.1 (-0.36 to 0.17) | 0.456 | -0.1 (-0.1 to 0.09) | <0.001 |
| Sex × Time | 0.51 (0.2 to 0.82) | 0.002 | 0.36 (0.35 to 0.36) | <0.001 |
|  | | | | |
| **Random Effects**: | | | | |
| σ² (Residual Variance) | <0.01 | | 0.01 | |
| τ₀₀ (ID Variance) | 0.07 ID | | 0.17 ID | |
| ICC (Intraclass Correlation) | 0.94 | | 0.94 | |
| N (Participants) | 27 ID | | 27 ID | |
| Marginal R² / Conditional R² | 0.305 / 0.956 | | 0.107 / 0.948 | |
|  | | | | |
| **Between Group Differences**: | | | | |
| **Time** | **Estimate (95% CI)** | **p** | **Estimate (95% CI)** | **p** |
| Month 1+ | 0.28 (-0.14 to 0.69) | 0.195 | 0.38 (0.37 to 0.39) | **<0.001** |
| Month 3+ | -0.23 (-0.65 to 0.19) | 0.279 | 0.02 (0.01 to 0.03) | **<0.001** |
|  | | | | |
| **Within Group Differences**: | | | | |
| **Group** | **Estimate (95% CI)** | **p** | **Estimate (95% CI)** | **p** |
| Male | 0.1 (-0.16 to 0.36) | 0.453 | 0.1 (0.09 to 0.11) | **<0.001** |
| Female | -0.41 (-0.56 to -0.25) | **<0.001** | -0.26 (-0.27 to -0.25) | **<0.001** |
| **Note:** Generalized Linear Mixed Models (GLMMs) fitted by maximum likelihood. | | | | |

**Supplemental Table 4: Comparisons of R2 nBR habituation change over time and between male and female patients with migraine with an interstimulus interval of 9s**

| **R² nBR Habituation** | **Interstimulus Interval 9s** | | | |
| --- | --- | --- | --- | --- |
|  | **Ipsilateral** | | **Contralateral** | |
| **Predictors** | **Estimate (95% CI)** | **p** | **Estimate (95% CI)** | **p** |
| (Intercept) | -0.31 (-0.33 to -0.29) | <0.001 | -0.23 (-0.69 to 0.23) | <0.001 |
| Sex | 0.03 (0.02 to 0.05) | 0.001 | -0.05 (-0.58 to 0.49) | 0.850 |
| Time | -0.04 (-0.05 to 0.02) | <0.001 | 0.03 (-0.42 to 0.48) | 0.899 |
| Sex × Time | 0.08 (0.06 to 0.1) | <0.001 | -0.07 (-0.59 to 0.45) | 0.786 |
|  | | | | |
| **Random Effects**: | | | | |
| σ² (Residual Variance) | 0.01 | | 0.01 | |
| τ₀₀ (ID Variance) | 0.03 ID | | 0.08 ID | |
| ICC (Intraclass Correlation) | 0.85 | | 0.9 | |
| N (Participants) | 27 ID | | 27 ID | |
| Marginal R² / Conditional R² | 0.044 / 0.858 | | 0.021 / 0.903 | |
|  | | | | |
| **Between Group Differences**: | | | | |
| **Time** | **Estimate (95% CI)** | **p** | **Estimate (95% CI)** | **p** |
| Month 1+ | -0.03 (-0.05 to -0.02) | **<0.001** | 0.05 (-0.47 to 0.57) | 0.849 |
| Month 3+ | -0.12 (-0.14 to -0.09) | **<0.001** | 0.12 (-0.4 to 0.65) | 0.649 |
|  | | | | |
| **Within Group Differences**: | | | | |
| **Group** | **Estimate (95% CI)** | **p** | **Estimate (95% CI)** | **p** |
| Male | 0.04 (0.02 to 0.05) | **<0.001** | -0.03 (-0.47 to 0.41) | 0.899 |
| Female | -0.04 (-0.07 to -0.02) | **0.001** | 0.04 (-0.22 to 0.3) | 0.747 |
| **Note:** Generalized Linear Mixed Models (GLMMs) fitted by maximum likelihood. | | | | |

**Supplemental Table 5: Comparisons of R2 nBR habituation change over time and between male and female patients with migraine with an interstimulus interval of 16s**

| **R² nBR Habituation** | **Interstimulus Interval 16s** | | | |
| --- | --- | --- | --- | --- |
|  | **Ipsilateral** | | **Contralateral** | |
| **Predictors** | **Estimate (95% CI)** | **p** | **Estimate (95% CI)** | **p** |
| (Intercept) | -0.24 (-0.56 to 0.07) | <0.001 | -0.17 (-0.6 to 0.25) | <0.001 |
| Sex | 0.31 (-0.06 to 0.67) | 0.103 | 0.19 (-0.3 to 0.68) | 0.447 |
| Time | 0 (-0.22 to 0.23) | 0.983 | 0.08 (-0.25 to 0.42) | 0.628 |
| Sex × Time | -0.05 (-0.32 to 0.22) | 0.711 | -0.15 (-0.54 to 0.24) | 0.432 |
|  | | | | |
| **Random Effects**: | | | | |
| σ² (Residual Variance) | <0.01 | | 0.01 | |
| τ₀₀ (ID Variance) | 0.05 ID | | 0.09 ID | |
| ICC (Intraclass Correlation) | 0.95 | | 0.94 | |
| N (Participants) | 27 ID | | 27 ID | |
| Marginal R² / Conditional R² | 0.232 / 0.959 | | 0.039 / 0.943 | |
|  | | | | |
| **Between Group Differences**: | | | | |
| **Time** | **Estimate (95% CI)** | **p** | **Estimate (95% CI)** | **p** |
| Month 1+ | -0.31 (-0.66 to 0.05) | 0.096 | -0.19 (-0.67 to 0.29) | 0.444 |
| Month 3+ | -0.26 (-0.61 to 0.1) | 0.165 | -0.03 (-0.51 to 0.45) | 0.892 |
|  | | | | |
| **Within Group Differences**: | | | | |
| **Group** | **Estimate (95% CI)** | **p** | **Estimate (95% CI)** | **p** |
| Male | 0 (-0.23 to 0.22) | 0.983 | -0.08 (-0.41 to 0.25) | 0.626 |
| Female | 0.05 (-0.09 to 0.19) | 0.504 | 0.07 (-0.12 to 0.27) | 0.470 |
| **Note:** Generalized Linear Mixed Models (GLMMs) fitted by maximum likelihood. | | | | |

**Supplemental Table 6: Comparisons of R2 nBR habituation change over time and between patients with and without aura with an interstimulus interval of 4s**

| **R² nBR Habituation** | **Interstimulus Interval 4s** | | | |
| --- | --- | --- | --- | --- |
|  | **Ipsilateral** | | **Contralateral** | |
| **Predictors** | **Estimate (95% CI)** | **p** | **Estimate (95% CI)** | **p** |
| (Intercept) | -0.85 (-1.08 to -0.63) | <0.001 | -0.61 (-0.95 to -0.26) | <0.001 |
| Aura | 0.03 (-0.38 to 0.45) | 0.871 | -0.26 (-0.88 to 0.37) | 0.413 |
| Time | 0.3 (0.12 to 0.48) | 0.002 | -0.01 (-0.22 to 0.21) | 0.967 |
| Aura × Time | -0.07 (-0.4 to 0.26) | 0.655 | 0.61 (0.2 to 1.02) | 0.004 |
|  | | | | |
| **Random Effects**: | | | | |
| σ² (Residual Variance) | 0.01 | | 0.01 | |
| τ₀₀ (ID Variance) | 0.07 ID | | 0.18 ID | |
| ICC (Intraclass Correlation) | 0.92 | | 0.95 | |
| N (Participants) | 27 ID | | 27 ID | |
| Marginal R² / Conditional R² | 0.217 / 0.940 | | 0.128 / 0.956 | |
|  | | | | |
| **Between Group Differences**: | | | | |
| **Time** | **Estimate (95% CI)** | **p** | **Estimate (95% CI)** | **p** |
| Month 1+ | -0.03 (-0.44 to 0.37) | 0.870 | 0.26 (-0.35 to 0.87) | 0.409 |
| Month 3+ | 0.04 (-0.37 to 0.45) | 0.847 | -0.35 (-0.98 to 0.28) | 0.275 |
|  | | | | |
| **Within Group Differences**: | | | | |
| **Group** | **Estimate (95% CI)** | **p** | **Estimate (95% CI)** | **p** |
| No Aura | -0.3 (-0.48 to -0.12) | **0.001** | 0.01 (-0.21 to 0.22) | 0.967 |
| Aura | -0.23 (-0.5 to 0.05) | 0.104 | -0.6 (-0.94 to -0.27) | **<0.001** |
| **Note:** Generalized Linear Mixed Models (GLMMs) fitted by maximum likelihood. | | | | |

**Supplemental Table 7: Comparisons of R2 nBR habituation change over time and between patients with and without aura with an interstimulus interval of 9s**

| **R² nBR Habituation** | **Interstimulus Interval 9s** | | | |
| --- | --- | --- | --- | --- |
|  | **Ipsilateral** | | **Contralateral** | |
| **Predictors** | **Estimate (95% CI)** | **p** | **Estimate (95% CI)** | **p** |
| (Intercept) | -0.34 (-0.53 to -0.16) | <0.001 | -0.31 (-0.59 to -0.03) | <0.001 |
| Aura | 0.21 (-0.13 to 0.55) | 0.219 | 0.13 (-0.37 to 0.64) | 0.607 |
| Time | 0.05 (-0.15 to 0.26) | 0.616 | -0.07 (-0.33 to 0.19) | 0.580 |
| Aura × Time | -0.1 (-0.49 to 0.29) | 0.600 | 0.17 (-0.33 to 0.68) | 0.495 |
|  | | | | |
| **Random Effects**: | | | | |
| σ² (Residual Variance) | <0.01 | | 0.01 | |
| τ₀₀ (ID Variance) | 0.03 ID | | 0.08 ID | |
| ICC (Intraclass Correlation) | 0.84 | | 0.9 | |
| N (Participants) | 27 ID | | 27 ID | |
| Marginal R² / Conditional R² | 0.168 / 0.864 | | 0.122 / 0.912 | |
|  | | | | |
| **Between Group Differences**: | | | | |
| **Time** | **Estimate (95% CI)** | **p** | **Estimate (95% CI)** | **p** |
| Month 1+ | -0.21 (-0.54 to 0.12) | 0.213 | -0.13 (-0.63 to 0.37) | 0.605 |
| Month 3+ | -0.11 (-0.44 to 0.22) | 0.518 | -0.31 (-0.8 to 0.19) | 0.231 |
|  | | | | |
| **Within Group Differences**: | | | | |
| **Group** | **Estimate (95% CI)** | **p** | **Estimate (95% CI)** | **p** |
| No Aura | -0.05 (-0.25 to 0.15) | 0.614 | 0.07 (-0.19 to 0.33) | 0.578 |
| Aura | 0.05 (-0.27 to 0.37) | 0.760 | -0.1 (-0.52 to 0.32) | 0.642 |
| **Note:** Generalized Linear Mixed Models (GLMMs) fitted by maximum likelihood. | | | | |

**Supplemental Table 8: Comparisons of R2 nBR habituation change over time and between patients with and without aura with an interstimulus interval of 16s**

| **R² nBR Habituation** | **Interstimulus Interval 16s** | | | |
| --- | --- | --- | --- | --- |
|  | **Ipsilateral** | | **Contralateral** | |
| **Predictors** | **Estimate (95% CI)** | **p** | **Estimate (95% CI)** | **p** |
| (Intercept) | -0.09 (-0.29 to 0.11) | <0.001 | -0.06 (-0.32 to 0.19) | <0.001 |
| Aura | 0.24 (-0.12 to 0.6) | 0.198 | 0.09 (-0.37 to 0.55) | 0.706 |
| Time | -0.02 (-0.16 to 0.13) | 0.822 | -0.09 (-0.28 to 0.12) | 0.409 |
| Aura × Time | -0.06 (-0.33 to 0.21) | 0.644 | 0.18 (-0.2 to 0.57) | 0.340 |
|  | | | | |
| **Random Effects**: | | | | |
| σ² (Residual Variance) | <0.01 | | 0.01 | |
| τ₀₀ (ID Variance) | 0.05 ID | | 0.09 ID | |
| ICC (Intraclass Correlation) | 0.95 | | 0.94 | |
| N (Participants) | 27 ID | | 27 ID | |
| Marginal R² / Conditional R² | 0.144 / 0.958 | | 0.089 / 0.944 | |
|  | | | | |
| **Between Group Differences**: | | | | |
| **Time** | **Estimate (95% CI)** | **p** | **Estimate (95% CI)** | **p** |
| Month 1+ | -0.24 (-0.59 to 0.12) | 0.192 | -0.09 (-0.54 to 0.36) | 0.705 |
| Month 3+ | -0.18 (-0.53 to 0.18) | 0.336 | -0.27 (-0.73 to 0.18) | 0.240 |
|  | | | | |
| **Within Group Differences**: | | | | |
| **Group** | **Estimate (95% CI)** | **p** | **Estimate (95% CI)** | **p** |
| No Aura | 0.02 (-0.12 to 0.16) | 0.821 | 0.09 (-0.11 to 0.28) | 0.405 |
| Aura | 0.08 (-0.15 to 0.3) | 0.491 | -0.1 (-0.42 to 0.22) | 0.537 |
| **Note:** Generalized Linear Mixed Models (GLMMs) fitted by maximum likelihood. | | | | |
